# Analysis of Factors Associated With Body Mass Index at Ages 18 and 36 Months Among Infants Born Extremely Preterm

**DOI:** 10.1001/jamanetworkopen.2021.28555

**Published:** 2021-10-14

**Authors:** Yayoi Murano, Hiromichi Shoji, Naho Ikeda, Natsuki Okawa, Kuniyoshi Hayashi, Masato Kantake, Naho Morisaki, Toshiaki Shimizu, Stuart Gilmour

**Affiliations:** 1 Department of Pediatrics, Juntendo University Faculty of Medicine, Hongo, Bunkyo-ku, Tokyo, Japan; 2 Graduate School of Public Health, St. Luke’s International University, Akashi-cho, Chuo-ku, Tokyo, Japan; 3 Department of Social Medicine, National Center for Child Health and Development, Okura, Setagaya-ku, Tokyo, Japan

## Abstract

**Importance:**

The development of neonatology has been associated with improved survival among infants born extremely preterm, and understanding their long-term outcomes is becoming increasingly important. However, there is little information on body mass index (BMI) among these children.

**Objective:**

To determine factors associated with BMI at ages 18 months and 36 months among infants born extremely preterm.

**Design, Setting, and Participants:**

This retrospective, multicenter cohort study was conducted using data from the Neonatal Research Network Japan database for 8838 infants born at gestational ages 23 to 28 weeks with data on BMI at 18 months and 36 months. Data were analyzed from April 2018 through June 2021.

**Exposures:**

BMI and BMI *z* score at ages 18 months and 36 months were regressed with gestational age, intrauterine growth restriction (IUGR) status, and complications during pregnancy and the neonatal period separately by presence of multiple pregnancy and sex.

**Main Outcomes and Measures:**

BMI and BMI *z* score at ages 18 months and 36 months.

**Results:**

Among 16 791 eligible infants born extremely preterm, 8838 infants were included in the analysis. There were 7089 infants born from single pregnancies (mean [SD] gestational age, 26.0 [1.6] weeks; 3769 [53.2%] boys; mean [SD] birth weight, 847 [228] g) and 1749 infants born from multiple pregnancies (mean [SD] gestational age, 26.3 [1.5] weeks; 903 [51.6%] boys; mean [SD] birth weight, 860 [217] g). In single pregnancies, every week of increased gestational age was associated with an increase in BMI of 0.21 (95% CI, 0.17-0.25) among boys and 0.20 (95% CI, 0.15-0.25) among girls at age 18 months and 0.21 (95% CI, 0.18-0.24) among boys and 0.21 (95% CI, 0.18-0.24) among girls at age 36 months. There was an interaction association between gestational age and IUGR among boys at age 36 months, with a decrease in the change associated with gestational age of 0.12 (95% CI, 0.05-0.19). Every week of increased gestational age in single pregnancies was associated with an increase in BMI *z* score of 0.14 (95% CI, 0.17-0.21) among boys and 0.17 (95% CI, 0.13-0.21) among girls at age 18 months and 0.19 (95% CI, 0.16-0.22) among boys and 0.17 (95% CI, 0.15-0.20) among girls at age 36 months. Among single pregnancies, IUGR was associated with a decrease in BMI among boys (0.59 [95% CI, 0.23-0.95]) and girls (0.75 [95% CI, 0.39-1.11]) and BMI *z* score among boys 0.85 [95% CI, 0.25-0.95)] and girls (0.67 [95% CI, 0.36-0.97] at age 18 months and BMI among boys (0.44 [95% CI, 0.17-0.18]) and girls (0.84 [95% CI, 0.55-1.12]) and BMI *z* score among boys (0.46 [95% CI, 0.21-0.71]) and girls (0.77 [95% CI, 0.53-1.01]) at age 36 months. In multiple pregnancies, IUGR was associated with a decrease in BMI *z* score at age 36 months among boys (0.26 [95% CI, 0.42-0.89]) and girls (0.29 [95% CI, 0.22-0.79]). In single pregnancies intraventricular hemorrhage (IVH) was associated with a decrease in BMI of 0.47 (95% CI, 0.21-0.73) among boys and 0.42 (95% CI, 0.13-0.71) among girls at age 18 months and 0.53 (95% CI, 0.32-0.74) among boys and 0.31 (95% CI, 0.07-0.54) among girls at age 36 months. IVH was associated with a decrease in BMI *z* score in single pregnancies of 0.63 (95% CI, 0.20-0.41) among boys and 0.35 (95% CI, 0.12-0.60) among girls at age 18 months and 0.53 (95% CI, 0.34-0.71) among boys and 0.30 (95% CI, 0.11-0.50) among girls at age 36 months. Similar associations were seen in multiple pregnancies.

**Conclusions and Relevance:**

This study found that gestational age, the presence of IUGR and multiple pregnancy, and IVH complications were associated with infant BMI at ages 18 months and 36 months. These findings suggest that these complicating factors should be considered when setting growth targets and nutrition strategies for infants born extremely preterm.

## Key Points

**Question:**

What factors are associated with body mass index (BMI) in the first 36 months of life among infants born at gestational age less than 28 weeks?

**Findings:**

In this cohort study of 8838 infants born extremely preterm, intrauterine growth restriction (IUGR) was associated with decreased BMI and BMI *z* score and older gestational age was associated with increased BMI and BMI z score at ages 18 months and 36 months among infants born in single pregnancies. In multiple pregnancies, IUGR was associated with decreased BMI *z* score at age 36 months, but the difference in scores was smaller.

**Meaning:**

This study found that among infants born extremely preterm, gestational age and the presence of IUGR were associated with subsequent BMI.

## Teaser

This cohort study investigates factors associated with body mass index in the first 36 months of life among infants born extremely preterm.

## Introduction

The survival rate of infants born preterm has improved dramatically during recent decades as neonatology has developed.^1^ The most notable change was in the 1980s with the development of pulmonary surfactant, which was associated with increased survival among infants born preterm with respiratory distress,^2^ and its clinical use was associated with a change in their clinical course.^3^ Other therapies, like ventilation techniques,^4^ antenatal glucocorticoid therapy, aggressive nutrition policies,^1^ and appropriate oxygen supplementation,^5^ are also still used. These developments were associated with improvements in the survival rate of infants born preterm^6^; from 1993 to 1980, the survival rate of infants in the US born weighing from 500 g to 550 g improved from 0% to 50% among Black male infants and from 35% to 70% among White female infants.^7^ In 2010, 90% of infants born very preterm (ie, birth weight <1500 g) in Germany survived.^8^ In Japan, one of the leading countries in neonatology, mortality rates among infants with very low birth weight at discharge improved from 2003 to 2008, with mortality decreasing from 10.8% to 8.7% among infants born with weights from 501 g to 750 g^9^ and decreases in mortality seen even among infants weighing less than 400 g.^10^

These developments in neonatology were associated with increased numbers of infants with short gestational age surviving their neonatal period,^5^ and their long-term health outcomes have become a matter of much concern. Understanding these outcomes is becomingly increasingly urgent.^11^ Studies among children born preterm have found different body shapes compared with children born at term,^12,13^ and differences in growth trajectories may be associated with various areas of cognitive function.^14,15^ However, the sample size of previous studies was small,^16,17,18^ with the largest a uniform sample size of 1320 infants.^19^ The mean gestational age of all samples in previous studies was at least 28 weeks,^20^ and there was little information about infants born at a gestational age of less than 30 weeks. Moreover, infants with intrauterine growth restriction (IUGR) were not separately analyzed in some studies.^21,22,23,24,25,26^ This condition results from uteroplacental insufficiency and is associated with chronic hypoxia and changes in all aspects of the growth process^27^ and pathological profile.^28^ Infants with IUGR are a high-risk population not only during their hospital stay,^29^ but also in their later lives, with increased risk of a wide range of health problems.^30,31^ It is important to include information about IUGR in studies of subsequent body shape, but to date this has not been done, to our knowledge. Moreover, given that many of the studies to date have been single-center studies, unmeasured center-specific factors may be associated with changes in the results.^32^ We conducted a large multicenter study of infants born extremely preterm (ie, at gestational age <28 weeks) to evaluate factors associated with body mass index (BMI; calculated as weight in kilograms divided by height in meters squared) at ages 18 months and 36 months.

## Methods

This cohort study was approved by the institutional review board of Tokyo Women's Medical University and registered as a prospective observational study with the University Hospital Medical Information Network. Written informed consent to the research and publication of the results was obtained from the parents or guardians of all infants. This study followed Strengthening the Reporting of Observational Studies in Epidemiology (STROBE) reporting guideline.

### Data Source

Data on extremely preterm births from January 1, 2003, to December 31, 2012, were extracted from the Neonatal Research Network of Japan (NRNJ) database. The NRNJ database was established in 2003 for clinical study by the Japan Society for Neonatal Health and Development, publicly released by NRNJ in 2016, and analyzed by us in 2018. The NRNJ database covers level 2 and level 3 newborn intensive care units (NICUs). A level 3 NICU is capable of caring for infants who are very small or very sick, and a level 2 NICU provides care to infants who weigh from 1200 g to 1800 g or were born from gestational age 30 weeks to 34 weeks. Every infant born alive and whose birth weight was less than 1500 g or who was born before gestational age 32 weeks was registered, and each participating NICU filled in a common questionnaire.

All infants born from gestational age 23 weeks to 28 weeks were eligible for this study. Infants were excluded from the study if they died during the hospital stay or follow up period, had unknown sex (ie, no information or unable to determine sex), or were born with a congenital anomaly. Measures of BMI were taken at corrected ages 18 months and 36 months. Body length or height and weight were measured using a scale for children without any clothing or shoes. Body length was measured with the child lying down until age 18 months, and at age 36 months, it was measured with the child standing.

### Data Collection

Information about birth weight, gestational age, death, parity, maternal complications during pregnancy (ie, multiple pregnancy and hypertension), complications during the infant’s hospital stay (ie, congenital anomaly, chronic lung disease [CLD], late circulatory collapse [LCC], intraventricular hemorrhage [IVH], necrotizing enterocolitis [NEC]), and body weight and height at ages 18 months and 36 months were extracted. The variables were selected using previous reports of factors associated with subsequent body composition.^33,34,35,36,37,38,39,40^ LCC is a form of hypotension that occurs after the early neonatal period with unclear underlying pathology that is common in Japan but not widely known in North America or Europe.^41^

We calculated BMI at ages 18 months and 36 months using weight and height. Then, using the LMS method and functions developed in previous research for Japanese infants,^42^ we calculated BMI *z* score. Until recently, there has been debate about the best tools for measuring body shape among infants. Recently, BMI has been reported to be a useful measurement among infants born preterm, and it is also reported to be a useful predictor associated with subsequent overweight status^43^ that is better associated with metabolic health outcomes.^44,45^ Furthermore, BMI curves among infants born preterm were published recently, and the reliability of BMI has been established in this field.^46^ Therefore, we presented BMI as a good score to inform long-term metabolic health outcomes among infants born very preterm. This score is also useful because it is easy to measure, which is an important consideration in health checkups.

### Statistical Analysis

First, to compare the characteristics of samples, we used *t* tests to compare BMI by the presence of IUGR. Then, we compared infant characteristics by the presence of multiple pregnancy. We used *t* tests for gestational age, birth weight, body length at birth, maternal age, and BMI. For proportion of sex, presence of IUGR, and parity more than 1, we used χ^2^ tests. We used the same procedure to examine differences between infants with data and those without data. Then, to understand features of complications during pregnancy and the neonatal period, we calculated the percentage of infants with complications, including pregnancy-induced hypertension, CLD, LCC, IVH, and NEC, separately by sex and presence of multiple pregnancy. Statistical tests were 1-sided, and results were considered statistically significant at *P* < .05 or when 95% CIs did not cross 0.

We analyzed single pregnancy and multiple pregnancy separately because in multiple pregnancy there is uterine overcrowding and growth rates and growth restriction patterns differ from single pregnancy.^47^ Additionally, we regressed BMI and BMI *z* score calculated at ages 18 months and 36 months against gestational age in weeks, with an interaction term for gestational age and IUGR because IUGR often leads to interventions of caesarian delivery or induced delivery.^48^ In this regression, non-IUGR status was set as the reference category. Gestational age was rescaled so that a value of 0 corresponded to gestational age 23 weeks so that the intercept term measured the BMI for an infant born without IUGR at gestational age 23 weeks. Parity, complications during pregnancy, and hospital stay, which were reported in previous studies^33,34,35,36,37,38,39,40^ to be associated with subsequent outcomes, were included as covariates. A backward stepwise model–building method was used to remove nonsignificant covariates.^49^ The analysis was performed using R statistical software version 4.0.3 (R Project for Statistical Computing). Data were analyzed from April 2018 through June 2021.

## Results

### Study Sample

Among 40 806 infants included in the NRNJ database, 19 510 infants born from gestational age 23 weeks to 28 weeks were eligible for this study. Among these, 2719 infants were excluded owing to unknown sex, death during the study period, or congenital anomaly, leaving 16 791 infants included in this study.

Among these infants, there were 7089 infants born from single pregnancies and 1749 infants born from multiple pregnancies available with data at least at ages 18 months or at 36 months in this study. Among these infants, 3783 infants from single pregnancies and 888 infants from multiple pregnancies had data at age 18 months, and 6258 infants from single pregnancies and 1554 infants from multiple pregnancies had data at age 36 months, including 2952 infants from single pregnancies and 693 infants from multiple pregnancies with data at both ages. Finally, 8838 infants (52.6%) were included in the sample, including 7089 infants from single pregnancies (80.2% of the study sample) and 1749 infants from multiple pregnancies (19.7% of the study sample).

Table 1 shows characteristics among infants born from single pregnancies compared with those born from multiple pregnancies. The mean (SD) gestational age was 26.0 (1.6) weeks among infants born from single pregnancies and 26.3 (1.5) weeks among children born from multiple pregnancies. The mean (SD) birth weight was 847 (228) g among single pregnancies and 860 (217) g among multiple pregnancies. There were 3769 [53.2%] boys born from single pregnancies and 903 [51.6%] boys born from multiple pregnancies. There were 1538 infants with IUGR (21.6%) among single pregnancies and 416 infants with IUGR (24.0%) among multiple pregnancies. In single pregnancies, gestational age, birth weight, and body length were lower. There were no differences in infant characteristics by sex or presence of IUGR. Mean (SD) BMI was increased among single pregnancies vs multiple pregnancies at ages 18 months (boys: 15.5 [1.4] vs 15.6 [1.3]; *P* < .001; girls: 15.2 [1.4] vs 15.1 [1.3]; *P* < .001) and 36 months (boys: 15.1 [1.3] vs 15.1 [1.4]; *P* < .001; girls: 14.9 [1.4] vs 14.8 [1.3]; *P* < .001). Mean (SD) BMI was increased among infants without IUGR vs infants with IUGR at ages 18 months (boys: 15.6 [1.3] vs 15.0 [1.3]; *P* < .001; girls: 15.4 [1.4] vs 14.6 [1.3]; *P* < .001) and 36 months (boys: 15.2 [1.3] vs 14.5 [1.2]; *P* < .001; girls: 15.1 [1.3] vs 14.3 [1.4]; *P* < .001) (Table 2). Prevalence of complications during pregnancy and the neonatal period ranged from a low for IVH, which occurred among 170 boys (4.5%) and 141 girls (4.3%) from single pregnancies and 56 boys (6.6%) and 42 girls (5.0%) from multiple pregnancies, to a high for CLD, which occurred among 2399 boys (64.0%) and 1960 girls (59.3%) from single pregnancies and 539 boys (60.0%) and 459 girls (54.3%) from multiple pregnancies (Table 3). In the eTable in the Supplement, characteristics among children with data and 7952 children without data are compared. We found no difference in the characteristics among infants with data vs those without data; the *P* values less than .05 were associated with the large the sample size, and there was little difference in the exact value of their characteristics. For example, mean (SD) gestational age was 26.1 (1.58) weeks among infants with data and 26.2 (1.59) weeks among infants without data (*P* = .04), while IUGR occurred among 1956 infants with data (22.1%) and 1607 (20.8%) infants without data (*P* = .01).

**Table 1.** Infant Characteristics by Single and Multiple Pregnancy

Characteristic^a^	Mean (SD)		*P* value
	Single pregnancy (n = 7089)	Multiple pregnancy (n = 1749)	
Gestational age, wk	26.0 (1.6)	26.3 (1.5)	<.001
Birth weight, g	847 (228)	860 (217)	<.001
Body length at birth, cm	33.1 (3.3)	33.5 (3.2)	.02
BMI available at follow-up period, No. (%)			
18 mo	3783 (53.3)	888 (50.7)	.15
36 mo	6258 (88.2)	1554 (88.9)	.79
Sex, No. (%)			
Boys	3769 (53.2)	903 (51.6)	.51
Girls	3320 (46.8)	846 (48.4)	
IUGR, No. (%)	1538 (21.6)	418 (24.0)	.14
Maternal age, y	31.5 (5.4)	30.9 (5.0)	<.001
Parity more than 1, No. (%)	3594 (50.7)	601 (34.4)	<.001

Abbreviations: BMI, body mass index (calculated as weight in kilograms divided by height in meters squared); IUGR, intrauterine growth restriction.

^a^ Gestational age, birth weight, body length at birth, and maternal age were evaluated using *t* test. The proportion of infants by sex and the proportion of infants with BMI available, IUGR, and parity more than 1 were evaluated by χ^2^ test.

**Table 2.** Comparison of BMI by Presence of IUGR

	BMI, mean (SD)		*P* value^a^
	With IUGR	Without IUGR	
At 18 mo			
Boys	15.0 (1.3)	15.6 (1.3)	<.001
Girls	14.6 (1.3)	15.4 (1.4)	<.001
At 36 mo			
Boys	14.5 (1.2)	15.2 (1.3)	<.001
Girls	14.3 (1.4)	15.1 (1.3)	<.001

Abbreviations: BMI, body mass index (calculated as weight in kilograms divided by height in meters squared); IUGR, intrauterine growth restriction.

^a^ Evaluated using *t* test.

**Table 3.** Complications During Pregnancy and Neonatal Period

Complication^a^	Infants, No. (%)			
	Single pregnancy (n = 7089)		Multiple pregnancy (n = 1749)	
	Boys (n = 3769)	Girls (n = 3320)	Boys (n = 903)	Girls (n = 846)
PIH	465 (12.3)	581 (17.6)	47 (5.2)	59 (7.0)
CLD	2399 (64.0)	1960 (59.3)	539 (60.0)	459 (54.3)
LCC	588 (15.8)	418 (12.7)	131 (14.8)	115 (13.7)
IVH	170 (4.5)	141 (4.3)	56 (6.6)	42 (5.0)
NEC	63 (1.7)	31 (0.9)	13 (1.5)	9 (1.1)

Abbreviations: CLD, chronic lung disease; IVH, intraventricular hemorrhage; LCC, late circulatory collapse; NEC, necrotizing enterocolitis; PIH, pregnancy-induced hypertension.

^a^ PIH was a maternal complication. Complications during the infant’s hospital stay included CLD, IVH, LCC, and NEC.

### BMI

Mean BMI was plotted for each gestational age with 95% CIs separately for boys and girls at ages 18 months and 36 months (Figure). At ages 18 months and 36 months, the mean BMI of infants with IUGR was decreased compared with those without IUGR at all gestational ages, with an increasing trend by gestational age in single pregnancies. However, this tendency was not present in multiple pregnancies. At age 18 months in single pregnancies, mean BMI was 14.5 (95% CI, 13.7-15.2) among boys with IUGR and 15.0 (95% CI, 14.8-15.2) among boys without IUGR and 13.9 (95% CI, 13.2-14.6) among girls with IUGR and 14.8 (95% CI, 14.5-15.0) among girls without IUGR born at gestational age 23 weeks; it was 15.2 (95% CI, 15.0-15.5) among boys with IUGR and 16.1 (95% CI, 15.9-16.2) among boys without IUGR and 14.9 (95% CI, 14.7-15.1) among girls with IUGR and 15.8 (95% CI, 15.7-16.0) among girls without IUGR born at gestational age 28 weeks. At age 36 months in single pregnancies, mean BMI was 14.2 (95% CI, 13.7-14.6) among boys with IUGR and 14.6 (95% CI, 14.4-14.7) among boys without IUGR and 13.6 (95% CI, 12.8-14.5) among girls with IUGR and 14.4 (95% CI, 14.2-14.6) among girls without IUGR born at gestational age 23 weeks; it was 14.7 (95% CI, 14.5-14.8) among boys with IUGR and 15.6 (95% CI, 15.1-15.8) among boys without IUGR and 14.6 (95% CI, 14.4-14.7) among girls with IUGR and 15.6 (95% CI, 15.5-15.8) among girls without IUGR born at gestational age 28 weeks.

**Figure.**
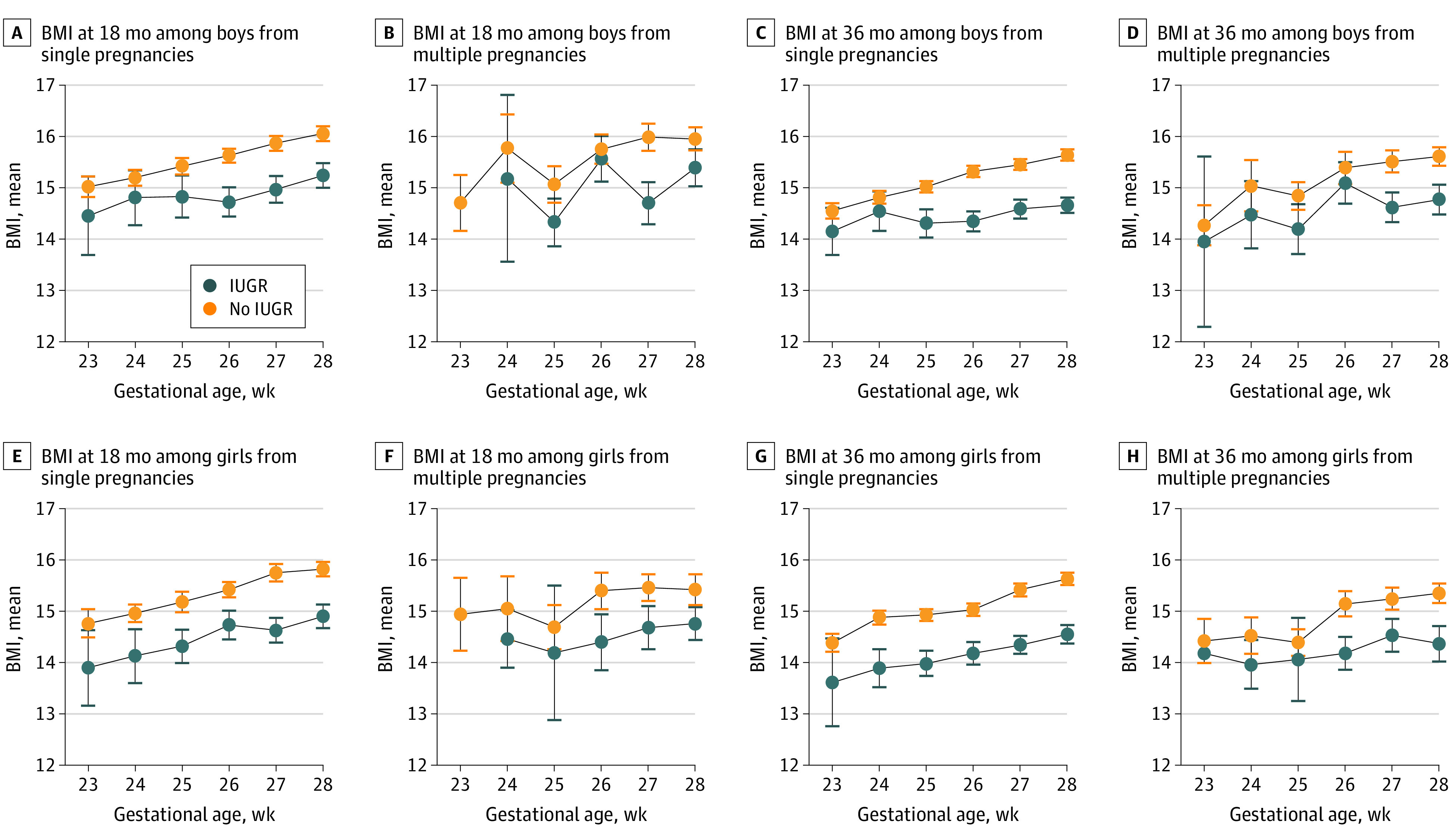
Body Mass Index (BMI) at Ages 18 Months and 36 Months by Sex and Multiplicity IUGR indicates intrauterine growth restriction; whiskers, 95% CIs.

### Linear Regression

We regressed BMI and BMI *z* score against gestational age in weeks with an interaction term for gestational age and IUGR, separately by sex and single vs multiple pregnancy. The coefficients and their 95% CIs are shown in Table 4.

**Table 4.** Linear Regression Analysis of BMI and BMI *z* Score by Pregnancy Number and Sex

	Coefficient (95% CI)	
	BMI	BMI *z* score
**Single pregnancies**		
At 18 mo		
Boys		
IUGR	−0.591 (−0.954 to −0.228)	−0.853 (−0.954 to −0.248)
GA	0.207 (0.167 to 0.247)	0.138 (0.171 to 0.205)
IVH	−0.472 (−0.731 to −0.214)	−0.626 (−0.196 to −0.411)
IUGR and GA (interaction)	−0.061 (−0.159 to 0.036)	−0.121 (0.040 to −0.040)
Girls		
IUGR	−0.753 (−1.114 to −0.392)	−0.666 (−0.967 to −0.364)
GA	0.204 (0.150 to 0.250)	0.170 (0.133 to 0.208)
LCC	−0.242 (−0.420 to −0.063)	−0.214 (−0.363 to −0.065)
IVH	−0.422 (−0.711 to −0.132)	−0.352 (−0.594 to −0.116)
IUGR and GA (interaction)	−0.047 (−0.146 to 0.052)	−0.029 (−0.112 to 0.054)
At 36 mo		
Boys		
IUGR	−0.443 (−0.178 to −0.167)	−0.461 (−0.711 to −0.212)
GA	0.210 (0.180 to 0.239)	0.189 (0.162 to 0.215)
IVH	−0.530 (−0.737 to −0.324)	−0.526 (−0.712 to −0.339)
IUGR and GA (interaction)	−0.120 (−0.194 to −0.045)	−0.096 (−0.163 to −0.028)
Girls		
IUGR	−0.836 (−1.123 to −0.549)	−0.768 (−1.008 to −0.528)
GA	0.209 (0.175 to 0.242)	0.174 (0.146 to 0.201)
LCC	−0.231 (−0.376 to −0.086)	−0.218 (−0.340 to −0.097)
IVH	−0.307 (−0.543 to −0.071)	−0.303 (−0.501 to −0.106)
IUGR and GA (interaction)	−0.049 (−0.127 to 0.029)	−0.029 (−0.094 to 0.036)
**Multiple pregnancies**		
At 18 mo		
Boys		
IUGR	−0.590 (−1.388 to 0.209)	−0.489 (−1.148 to 0.171)
GA	0.169 (0.083 to 0.255)	0.144 (0.073 to 0.215)
IVH	−0.970 (−1.438 to −0.502)	−0.840 (−1.226 to −0.454)
IUGR and GA (interaction)	−0.061 (−0.159 to 0.036)	−0.027 (−0.195 to 0.141)
Girls		
IUGR	−0.712 (−1.675 to 0.251)	−0.604 (−1.412 to 0.204)
GA	0.121 (0.027 to 0.216)	0.103 (0.023 to 0.182)
IVH	−0.705 (−1.257 to −0.153)	−0.639 (−1.102 to −0.175)
IUGR and GA (interaction)	−0.005 (−0.246 to 0.237)	0.001 (−0.202 to 0.204)
At 36 mo		
Boys		
IUGR	−0.320 (−0.907 to 0.268)	−0.257 (−0.890 to −0.422)
GA	0.180 (0.109 to 0.251)	0.169 (−0.770 to 0.256)
LCC	−0.422 (−0.689 to −0.154)	−0.416 (−0.650 to −0.182)
IVH	−1.017 (−1.399 to −0.636)	−0.996 (−1.329 to −0.663)
NEC	−0.968 (−1.717 to −0.219)	−0.941 (−1.595 to −0.286)
IUGR and GA (interaction)	−0.111 (−0.264 to 0.042)	−0.104 (−0.238 to 0.030)
Girls		
IUGR	−0.753 (−0.951 to −0.224)	−0.287 (−0.792 to −0.218)
GA	0.205 (0.137 to 0.268)	0.175 (−0.119 to 0.232)
LCC	−0.242 (−0.548 to −0.020)	−0.261 (−0.487 to −0.034)
IVH	−0.422 (−0.845 to −0.023)	−0.478 (−0.831 to −0.126)
IUGR and GA (interaction)	−0.047 (−0.271 to 0.039)	−0.107 (−0.240 to 0.026)

Abbreviations: GA, gestational age; IUGR, intrauterine growth restriction; IVH, intraventricular hemorrhage; LCC, late circulatory collapse; NEC, necrotizing enterocolitis.

Among single pregnancies, gestational age and IUGR were associated with BMI at age 18 months among boys (gestational age: coefficient, 0.21 [95% CI, 0.17 to 0.25]; IUGR: coefficient, −0.59 [95% CI, −0.95 to −0.23]) and girls (gestational age: coefficient, 0.20 [95% CI, 0.15 to 0.25]; IUGR: coefficient, −0.75 [95% CI, −1.11 to −0.39]). Gestational age and IUGR were also associated with BMI at age 36 months in single pregnancies among boys (gestational age: coefficient, 0.21 [95% CI, 0.18 to 0.24]; IUGR: coefficient, −0.44 [95% CI, −0.18 to −0.17]) and girls (gestational age: coefficient, 0.21 [95% CI, 0.18 to 0.24]; IUGR: coefficient, −0.84 [95% CI, −1.12 to −0.55]), and there was an interaction association between gestational age and IUGR among boys (coefficient, −0.12 [95% CI, −0.19 to −0.05]) at age 36 months. In multiple pregnancies, IUGR was not associated with BMI, except among girls at age 36 months (coeffecient, −0.75 [95% CI, −0.95 to −0.22). Gestational age in multiple pregnancies was associated with BMI at age 18 months among boys (coefficient, 0.17 [95% CI, 0.08 to 0.26]) and girls (coefficient, 0.12 [95% CI, 0.03 to 0.22]) and at age 36 months among boys (coefficient, 0.18 [95% CI, 0.11 to 0.25]) and girls (coefficient, 0.21 [95% CI, 0.14 to 0.27).

Gestational age and IUGR were also associated with BMI *z* score in single pregnancies at age 18 months among boys (gestational age: coefficient, 0.14 [95% CI, 0.17 to 0.21]; IUGR: coefficient, −0.85 [95% CI, −0.95 to −0.25)]) and girls (gestational age: coefficient, 0.17 [95% CI, 0.13 to 0.21]; IUGR: coefficient, −0.67 [95% CI, −0.97 to −0.36]) and age 36 months among boys (gestational age: coefficient, 0.19 [95% CI, 0.16 to 0.22]; IUGR: coefficient, −0.46 [95% CI, −0.71 to −0.21]) and girls (gestational age: coefficient, 0.17 [95% CI, 0.15 to 0.20]; IUGR: coefficient, −0.77 [95% CI, −1.01 to −0.53]). However, in multiple pregnancies, gestational age was associated with BMI *z* score at age 18 months among boys (coefficient, 0.14 [95% CI, 0.07 to 0.22]) and girls (coefficient, 0.10 [95% CI, 0.02 to 0.18]), while IUGR was associated with BMI *z* score at age 36 months among boys (coefficient, −0.26 [95% CI, −0.89 to −0.42]) and girls (coefficient, −0.29 [95% CI, −0.79 to −0.22]).

Birth complications associated with BMI in single pregnancies included IVH among boys at age 18 months (coefficient, −0.47 [95% CI, −0.73 to −0.21]) and 36 months (coefficient, −0.53 [95% CI, −0.74 to −0.32]), LCC among girls at age 18 months (coefficient, −0.24 [95% CI, −0.42 to −0.06]) and 36 months (coefficient, −0.23 [95% CI, −0.38 to −0.09]), and IVH among girls at age 18 months (coefficient, −0.42 [95% CI, −0.71 to −0.13]) and 36 months (coefficient, −0.31 [95% CI, −0.54 to −0.07]). Among single pregnancies, IVH was associated with BMI *z* score among boys (coefficient, −0.63 [95% CI, −0.20 to −0.41) and girls (−0.35 [95% CI, −0.60 to −0.12) at age 18 months and among boys (−0.53 [95% CI, −0.71 to −0.34) and girls (−0.30 [95% CI, −0.50 to −0.11) among girls at age 36 months. Among multiple pregnancies, birth complications associated with BMI included IVH among boys (coefficient, −0.97 [95% CI, −1.44 to −0.50]) and girls (coefficient, −0.71 [95% CI, −1.26 to −0.15]) at age 18 months; LCC (coefficient, −0.42 [95% CI, −0.69 to −0.15]), IVH (coefficient, −1.02 [95% CI, −1.40 to −0.64]), and NEC (coefficient, −0.97 [95% CI, −1.72 to −0.22]) among boys at age 36 months; and LCC (coefficient, −0.24 [95% CI, −0.55 to −0.02]) and IVH (coefficient, −0.42 [95% CI, −0.85 to −0.02]) among girls at age 36 months.

## Discussion

In this cohort study, we collected data on infants born very preterm from the NRNJ database. This is the largest database of infants hospitalized in NICUs in Japan that is suitable for research. We found that gestational age and the presence of IUGR at birth were associated with BMI and BMI *z* score at ages 18 months and 36 months.

To the best of our knowledge, this was the first study of BMI among infants born very preterm to simultaneously consider gestational age, IUGR, and multiple pregnancy in a large, multicenter sample. Our findings suggest that it is no longer reasonable to discuss BMI without considering gestational age and IUGR information among infants born very preterm. Previous studies of infant and childhood BMI among individuals born extremely preterm had small sample sizes,^19,20^ nonuniform samples,^21^ or more mature samples with analysis by categories of gestational age.^17,18,24^ Moreover, while infants born with IUGR should be analyzed separately because this condition is associated with delays in all aspects of the growth process,^20,27^ many complications,^28^ and increased risk of long-term health problems,^29,30,31^ many previous studies did not account for this condition.^21,24,25^ Similarly, in our study, we analyzed singleton and multiple pregnancies given that multiple pregnancies are associated with a different prognosis,^50,51,52^ pathology, and cause of prematurity,^53,54^ while this was not done in previous studies, to our knowledge.

Because of the large sample size and hospital coverage in this study, we could adjust for confounding factors from pregnancy complications, finding that children experiencing IVH had decreased BMI at ages 18 months and 36 months compared with those not experiencing IVH. This condition may be associated with growth outcomes^55^ through cerebral palsy, disability, cognitive impairment,^40^ and feeding disorders.^56,57^ Nutritional interventions have been studied among infants born preterm with IVH,^58^ and our study findings support the importance of these outcomes for long-term development among these children.

Most previous studies of preterm birth^11,59,60,61^ have focused on neurological or developmental outcomes, and there are few guidelines on body weight measurement or tools for understanding growth patterns among infants born extremely preterm. Our results may be a useful guide for caregivers.

Our study found decreased BMI at ages 18 months and 36 months among infants born preterm. Although extrauterine growth restriction is a well-known complication at discharge among infants born preterm that is associated with cardiometabolic outcomes and neurodevelopmental outcomes,^62^ the causes are unknown.^63^ However, infants born preterm are already small at discharge. So far, studies have suggested that gastroesophageal reflux,^64^ eating disorders,^65^ sleeping disorders,^66^ and abnormal fat distribution^26^ are associated with growth among infants born preterm. However, infants born preterm grow in a different environment than the uterus, and epigenetic factors are reported to be associated with growth outcomes,^67,68^ but further investigation is required from this perspective.

### Limitations

This study has several limitations. The first limitation was the large number of infants with no data. This could be because caretakers of infants who had good prognoses stopped bringing them to the hospital for health checkups. However, we found no difference in the characteristics among infants with data vs those without data. The second limitation was the lack of information on nutritional status, which prevented us from adjusting for the confounding interaction of nutritional interventions with growth. The third limitation was that there was a small number of infants with decreased gestational age, especially in multiple pregnancies, that may be associated with survivor bias. However, despite this small number, ours is the first study, to our knowledge, to assess growth outcomes among these infants born very preterm and may offer important first insights into their subsequent growth curve. The fourth limitation was that we did not determine severity of IUGR, just presence or absence of this condition. However, in our clinical practice, we use the presence of IUGR rather than severity as a clinical marker, so understanding the long-term outcomes associated with IUGR may be more important clinically than assessing the associations between severity of growth restriction and BMI.

## Conclusions

Given that more infants born preterm are surviving their perinatal period and their survival rate continues to improve,^9^ it is important to know their long-term outcomes. Japan is at the forefront of neonatology,^10^ and revealing the subsequent features of infants born preterm in this country may contribute to neonatology in other countries. This study found that among infants born before gestational age 28 weeks, gestational age was associated with increased subsequent BMI and the presence of IUGR was associated with decreased BMI among infants born from single pregnancies. These findings suggest that when we follow up with these infants, we should be aware of their baseline characteristics and be conscious of the difficulty in gaining weight in this high-risk population. As more infants survive at younger gestational ages, policy developed using the findings of this study may help ensure they are able to thrive to adulthood and enjoy the full benefits of their survival.

## Notes

Supplement. **eTable.** Characteristics Among Children With Data and Without Data
