# Supplementary material for: Analysis of Factors Associated With Body Mass Index at Ages 18 and 36 Months Among Infants Born Extremely Preterm
Source: JAMA Netw Open. 2021 Oct 14;4(10):e2128555. doi: 10.1001/jamanetworkopen.2021.28555 (PMC8517745; doi:10.1001/jamanetworkopen.2021.28555)
Supplement: Supplement. — eTable. Characteristics Among Children With Data and Without Data [file jamanetwopen-e2128555-s001.pdf]

Supplemental Online Content

Murano Y, Shoji H, Ikeda N, et al. Analysis of factors associated with body mass index at ages 18 and 36 months among infants born extremely preterm. *JAMA Netw Open*. 2021;4(10):e2128555. doi:10.1001/jamanetworkopen.2021.28555

eTable. Characteristics Among Children With Data and Without Data

This supplemental material has been provided by the authors to give readers additional information about their work.

© 2021 Murano Y et al. *JAMA Network Open.*

eTable. Characteristics Among Children With Data and Without Data

Variable	18- or 36-month data available (8838)	Neither 18- nor 36-month data available (7952)	P
Gestational age (weeks)	26.1 ± 1.58	26.2 ± 1.59	0.04
Birth weight at birth (g)	850.2 ± 226.1	869.8 ± 229.7	< 0.05
Body length at birth (cm)	33.2 ± 3.3	33.4 ± 3.2	< 0.05
IUGR (%)	1956 (22.1)	1607 (20.8)	< 0.05
Maternal age	31.3 ± 5.3	30.9 ± 5.5	< 0.05
Multiple pregnancy (%)	1749 (19.8)	1686 (21.2)	0.08
Sex (boys) (%)	4672 (52.9)	4203 (52.9)	1.00
Parity more than 1 (%)	4195/ 8785 (47.8)	3873/ 7808 (49.6)	0.06
PIH (%)	1152/ 8803 (13.1)	904/ 7809 (11.6)	0.013
LCC	1252/ 8742 (14.3)	967/ 7774 (12.4)	0.002
CLD	5357/ 8797 (60.9)	4612/ 7787 (59.2)	0.09
NEC	116/ 8755 (1.3)	155/ 7802 (2.0)	0.004
IVH	1605/ 8798 (18.2)	1473/ 7831 (18.8)	0.64

© 2021 Murano Y et al. *JAMA Network Open.*
